# Expenditures on sugar-sweetened beverages in Jamaica and its association with household budget allocation

**DOI:** 10.1186/s12889-022-12959-7

**Published:** 2022-03-24

**Authors:** Guillermo Paraje, Fabio S. Gomes

**Affiliations:** 1 Escuela de Negocios, Universidad Adolfo Ibáñez, Santiago de Chile, Chile; 2 Pan American Health Organization/World Health Organization Regional Office for the Americas, Washington, DC USA

**Keywords:** Jamaica, Sugar-sweetened beverages, Budget allocation

## Abstract

**Background:**

Sugar-sweetened beverages (SSB) consumption is associated with overweight and obesity, which are important drivers for the increasing healthcare and other social costs. If expenditures on SSB decrease expenditures on other goods and services, such as education and healthcare, this “crowding-out” may have a lasting effect. The main objectives of this article are, first, to estimate the statistical association between the decision of spending in SSB and several households’ sociodemographic characteristics; and second, to estimate the association between the decision of buying SSB and budget allocation across categories in Jamaica.

**Methods:**

Using the Jamaican Household Expenditure Survey 2004–2005 a generalized ordered probit model was estimated to examine the association between socioeconomic variables and the decision to spend on SSB. Seemingly Unrelated Regression Equations (SURE) of all the expenditure groups (except the SSB group) were used to estimate the association between the decision of buying SSB and budget allocation on other goods and services.

**Results:**

Expenditures on SSB are negatively affected by the size of the household and the area of residence (rural households spend more on SSB than urban ones), while having a larger proportion of children (15 or younger) and having a larger total budget is associated to more expenditures on SSB. Households with positive expenditure on SSB allocate significantly less budget to “Healthcare” and “Education”, when compared to those who did not buy SSB.

**Conclusions:**

SSB expenditures may displace expenditures in necessary goods and services, which implies that decreasing the proportion of budget spent on SSB may have important present and future consequences on poorer households’ human capital accumulation and future incomes.

**Supplementary Information:**

The online version contains supplementary material available at 10.1186/s12889-022-12959-7.

## Background

According to a 2018 World Health Organization Global report, obesity has nearly tripled between 1975 and 2016 and, globally, more than 1.9 billion people aged 18 and older were overweight, with more than 650 million considered obese in 2016 [1]. Among children the overweight/obesity epidemics is also growing: in 2016, 340 million children and adolescents aged 5–19 years, and around 40 million children under the age of 5 years, were considered overweight or obese [1]. The region of the Americas has the highest prevalence of adult obesity in the world, with 28.6%, which is more than double the global prevalence of 13.1% [1].

In the case of Jamaica, the prevalence of adults with overweight doubled from 27.4% in 1975 to 55.5% in 2016, while the prevalence of obesity among adults increased from 6.9% in 1975 to 24.7% in 2016, nearly a fourfold increase [2]. For childhood obesity the situation is even worse. Its prevalence increased from 1% in 1975 to 13% in 2016, thirteen times greater, with an average annual increase of 6.3% [2].

Increases in overweight and obesity have been associated with a number of health conditions, including the most burdensome ones such as cardiovascular diseases, different types of cancer, and diabetes [3]. Prominent experts in public health have signalled the consumption of sugar-sweetened beverages (SSB) as “the single largest driver of the obesity epidemic”, calling for extensive taxation and regulation of such products [4]. In addition to its impact on overweight/obesity, the scientific evidence relating the consumption of SSB to negative health outcomes is vast and has been accumulating over the last decade. A 2007 systematic review found that SSB consumption was associated with an increase in caloric intake, beyond the levels contributed by the said beverages and an increase in body weight [5]. In addition, it found negative associations (moderate but significant) between the consumption of SSB and certain nutritious foods like milk and essential nutrients like calcium. There is also evidence of a positive relationship between SSB consumption and cardiovascular diseases and type 2 diabetes mellitus [6–9]. These associations points toward a significant increase in future health system costs associated with overweight/obesity and noncommunicable diseases, in relation to SSB consumption [10, 11].

A recent study measured global, regional and national consumptions of SSB and milk [12]. It has demonstrated that the average SSB intake in Jamaica is 3.29 and 3.58 servings/day (serving = 8 oz = 237 ml) for female and male (aged 20–30 years), respectively. These figures are more than three times higher than the global average intake for female and male population with similar age range (20–39 years): 0.94 and 1.04 servings/day, respectively. Another, related study, found that, in 2010, more than 380 people (over 20 years old) died in Jamaica from diseases directly attributed to SSB consumption (4.8% of all deaths among that group). The vast majority of these deaths were related to diabetes [13].

Overweight and obesity are also important drivers for the increasing healthcare costs. Direct healthcare costs increase because of the many conditions that are caused by overweight/obesity. Other indirect, usually much higher costs are also present, such as loss of human capital, job absenteeism and presenteeism, disability pension, loss of quality-life years, and premature deaths [14]. Family costs, such as the cost of suffering and loss earnings for caregivers, can also be significant and greatly increase total social costs, though they are often difficult to measure. Studies measuring such costs for low- and middle-income countries are scarce, though a recent study conducted in Chile, Ecuador and Mexico found that direct and indirect costs for overweight/obesity may range from 0.2% of GDP (Chile) to 1.7% of GDP (Ecuador) with important increases expected in the near future [15]. In the case of Jamaica and other Caribbean countries, although there are no reasons to expect they would be different from the abovementioned three countries, there are no studies quantifying such costs.

Apart from present social costs, consumption of SSB may influence future outcomes, from family future incomes to national economic growth. If expenditures on SSB decrease expenditures on other goods and services, such as education and healthcare, this “crowding-out” may have a lasting effect. Families in such a situation may end up with lower human capital accumulated, which could imply lower earnings and higher healthcare costs in the future [16]. At the aggregate level, lower human capital is related to permanent lower economic growth [17].

Although a number of studies have analyzed the effect that consumption of unhealthy products (e.g. tobacco) has on the allocation of households expenditures [18, 19], such analysis has not been conducted for SSB in the case of Jamaica. The objectives of this article are, first, to estimate the statistical association between the decision of spending in SSB and several households’ sociodemographic characteristics; and second, to estimate the association between the decision of buying SSB and budget allocation across categories in Jamaica. Then, policy recommendations to reduce the disease burden associated with SSB consumption are discussed.

Two types of analyses are conducted. The first one seeks to shed light on what socio-demographic variables are related to having expenditures on SSB according to households’ levels of SSB expenditures. The second analysis seeks to establish the statistical association between expenditure allocation on SSB and on other goods and services. Given their fixed budget, households must decide on how to allocate it and therefore in which goods and services they would spend it on.

## Methods

Data for the analyses come from the Jamaican Household Expenditure Survey 2004 – 2005 (HES 2004 – 2005), which was collected between June 2004 and March 2005 by the Statistical Institute of Jamaica [20]. The HES 2004 – 2005 used a two-stage stratified random sample design, where the first stage was the selection of Primary Sampling Units (PSUs). In the second stage, a number of households from each PSUs were selected. A total of 12,012 households were selected to be surveyed over a period of ten months, and the response rate was of 73.8%, totaling 8,865 households with a completed survey. There is no information on the characteristics of non-responding households. However, comparing the proportional size of certain population groups (e.g., ages 15 and over; ages 65 and over; etc.) from the survey with estimates from the United Nations [21], there are no differences or they are negligible. This may indicate that the resulting sample is representative of the Jamaican population. A total of 233 households (2.6% of the total sample) do not have complete information on expenditures (the sum of expenditures in all categories is less than the total household expenditures) and were discarded. The removal of these observations from the statistical analyses do not significantly change results (See Table S2 in in the Supplementary material).

Data contains information on all categories of expenditures; employment status of all members of the household; household incomes; personal and household characteristics, such as age, gender, area of residence (urban/rural), household size, etc. For the sake of the analyses, household expenditures were classified into 16 categories: 1) Food (consumed at home); 2) Tea, coffee and cocoa; 3) SSB; 4) Water (non-SSB); 5) Alcoholic beverages; 6) Tobacco; 7) Clothing and Footwear; 8) Housing, water, electricity, gas and other fuels; 9) Furnishings, household equipment and routine household maintenance; 10) Healthcare; 11) Transport; 12) Communication; 13) Recreation and culture; 14) Education; 15) Restaurants and hotels; 16) Others.

Every category, except the first four ones, follows the Classification of Individual Consumption According to Purpose (COICOP) classification [22]. The first group of COICOP classification was further divided into four subgroups "Food (consumed at home)", “Tea, coffee and cocoa”, "SSB" (carbonated beverages -bottled or canned-, nectars and juices -bottled, canned, boxed- of several flavors, etc.) and "Water" to perform the analysis. The COICOP groups of “Miscellaneous goods and services”, “Taxes” and “Donations” were grouped in the category specified as “Others”. The exact codes considered for each category are mentioned in Table S1 in the Supplementary material.

Two different models are estimated to characterize households’ decisions related to SSB expenditures. First, a generalized ordered probit model (GOPM) was estimated to examine the association between the decision to spend on SSB and socioeconomic variables. The dependent variable is ordinal and takes four possible values: 0 if the household does not purchase SSB; 1 if household spend a “low amount” on SSB; 2 if they spend a “medium amount” on SSB; and 3 if they spend a “high amount” on SSB. Categories of expenditures on SSB are *ad-hoc* and constructed using tertiles (33% of the distribution) of the total household expenditure on SSB. GOPM are more parsimonious than probit models when data is ordered [23], as it is this case. In addition, GOPM do not have to satisfy the parallel lines assumption that ordered probit models (OPM) have to satisfy [23]. A likelihood-ratio test, testing the parallel lines assumption, is conducted to choose between them [24]. The likelihood-ratio test to test the parallel lines assumption is rejected at a significance of 1% (results not shown but available form authors). Hence, the GOPM is preferred over the OPM.

The functional form for these models has been described in detail elsewhere [24]. In our case, the independent variables include the area of residence of the household (urban or rural); the sex, age and age squared of the head of the household; the natural logarithm of the household size; the proportion of women in the household; the proportion of children in the household (younger than 15); a dichotomous variable taking the value of one if there is at least one employed member in the household; and the natural logarithm of total household expenditure.

The second model estimates the statistical association between the decision of buying SSB and the budget allocation on other goods and services. This is a reduced form based on a model of households’ utility maximization subject to a budget restriction, that assumes that households determine what proportion of their budget they would first allocate to a certain product (e.g. SSB) and then determine the proportion allocated to other budget categories and, subsequently, products. In this case, it is important to determine if the product first considered in the budget allocation is weakly separable from the consumption of the other products. Weak separability would imply that consumption of such a product only generates an income effect (it only decreases the absolute consumption of other products because of the lower budget net of the expenditures in such a product) and does not have a substitution effect (the consumption of such a product does not change the marginal substitution rate among the rest of the products) [25, 26]. In practical terms, weak separability would imply that households purchasing SSB would have the same budget allocation than comparable households not purchasing SSB, if faced with similar market conditions.

The assumption of weak separability of SSB can be considered by estimating a system of equations in which each individual equation, takes the form:

$${w}_{ih}=\alpha +\beta \cdot {SSB}_{h}+\gamma \cdot {X}_{h}+{\varepsilon }_{ih}$$

where ${w}_{ih}$ is the share of the household total expenditures allocated to the good/service *i* by the household *h*, where *i* can be any of the categories of goods/services specified; SSB is a dichotomous variable that takes the value of one if the household *h* has positive expenditure on SSB and ${X}_{h}$ are a set of sociodemographic variables for household *h*. These are the same variables included in the vector *X* for the GOPM. Because budget shares of the budget categories may add up to one (adding up restriction), one category must be arbitrarily dropped [25]. In this case, we drop the “Other” category and estimate a system with 14 equations (the 16 categories defined above, except for “SSB” and “Others”).

A positive (negative) *β* for category *i* means that the purchase of SSB is associated with an increase (decrease) in the share of expenditures devoted to that category of goods/services. On the other hand, a negative coefficient indicates that spending on SSB is related to a decision to spend less in that category of goods/services.

Because the decision of households to allocate their budget is made simultaneously, the system of equations may have errors (${\varepsilon }_{ih}$) that are correlated (contemporaneous correlation). To contemplate this, the system was estimated using Seemingly Unrelated Regression Equations (SURE), as recommended in similar studies [19, 25–28]. The SURE estimation allows to estimate a system of equations where the errors of each equation may be correlated to the errors of the other equations, an assumption that is reasonable in a context where budget allocations are made (almost simultaneously), given a certain budget restriction [29].

Using SURE with the same independent variables across equations results in coefficients that are the same and can be interpreted as those obtained from a set of Ordinary Least Square (OLS) regressions, estimated independently. When estimating the same SURE but for the budget shares of “*Housing, Water, Electricity, *etc*.”; “Furnishing, household equipment, *etc*.”; and “Communication”*, not using as regressors the log of household size, the proportion of women and the proportion of children 15 and younger (because in those expenditures there are intra-household economies of scale in consumption), the results (shown in Table S3 in the Supplementary material) remain qualitatively unchanged.

The estimation of both models considers the structural information of sample design and sampling weights. Models are estimated using Stata 16.1/MP. All methods were performed in accordance with the relevant guidelines and regulations.

## Results

Table 1 displays sociodemographic characteristics of the sample according to SSB expenditures (no expenditures and positive expenditures), while Table 2 presents the expenditure structure of the households disaggregated by the same groups as Table 1. Assuming households do not make stocks of SSB neither waste them, SSB purchases are equal to SSB consumption. Table 1 shows that 68% of the households registered positive expenditure on SSB. Households with higher consumption present a higher percentage of urban households (59%) compared to those with low or no consumption (50%). Households that did not have SSB expenditures reported a lower percentage of children (26.1%) than those with positive SSB expenditures (27.7%). The average total annual expenditure of households was $478,456 Jamaican dollars (with a standard deviation of $434,317), equivalent to between 12,686 and 14,120 international dollars, using the Power Purchasing Parity -PPP- conversion factor for private consumption, for 2004 and 2005 [30]). Such an amount was higher in the households that spends on SSB (an annual amount of $539,001 with a standard deviation of $460,664; versus $349,544 in households with no SSB expenditures with a standard deviation of $337,713). Private consumption refers, in this context, to household final consumption expenditure [30].

**Table 1:** Mean and standard deviation of sociodemographic variables

Variable	No SSB Expenditures	Positive SSB Expenditures^a^	Low SSB Expenditures	Medium SSB Expenditures	High SSB Expenditures	Total^b^
% of households with positive expenditures SSB	0.00%	100.00%	100.00%	100.00%	100.00%	68.04%
Standard Deviation	0.00	0.00	0.00	0.00	0.00	0.47
% of households with positive expenditures on water (non SSB)	52.34%	47.66%	53.21%	49.42%	43.07%	12.40%
Standard Deviation	0.50	0.50	0.50	0.50	0.50	0.33
% of female head of the household	42.02%	43.47%	44.73%	44.24%	41.42%	43.01%
Standard Deviation	0.49	0.50	0.50	0.50	0.49	0.50
% of urban households	50.12%	54.73%	50.39%	54.86%	59.03%	53.25%
Standard Deviation	0.50	0.50	0.50	0.50	0.49	0.50
Average age of household head	49.85	46.68	47.54	46.56	45.91	47.69
Standard Deviation	16.66	16.15	16.68	15.82	15.87	16.38
Average household Size	3.38	3.50	3.42	3.46	3.61	3.46
Standard Deviation	2.26	2.31	2.24	2.30	2.39	2.30
% of Women in household	46.53%	47.80%	48.48%	47.89%	47.00%	47.39%
Standard Deviation	0.32	0.31	0.32	0.31	0.30	0.32
% of children in the household (< 18)	26.13%	27.74%	27.84%	27.14%	28.22%	27.23%
Standard Deviation	0.27	0.26	0.27	0.26	0.25	0.26
Number of employed in the household	1.12	1.19	1.11	1.20	1.25	1.17
Standard Deviation	0.92	0.94	0.93	0.94	0.94	0.93
Total annual expenditures	$349,544	$539,001	$391,259	$508,598	$719,724	$478,456
Standard Deviation	337,713	460,664	376,127	410,348	521,135	434,317

Low, medium and high SSB consumption are tertiles of households grouped according to positive expenditures on SSB

^a^Positive expenditures include all households that have some expenditures on SSB

^b^“Total” includes all households in the sample (8,632 households)

**Table 2:** Mean and standard deviations of budget shares by goods/services groups

Variable	No SSB Expenditure	Positive SSB Expenditure^a^	Low SSB Expenditure	Medium SSB Expenditure	High SSB Expenditure	Total^b^
Budget share in food (at home)	37.81%	35.06%	34.63%	34.93%	35.61%	35.94%
SD	0.20	0.17	0.19	0.17	0.16	0.18
Budget share in tea, coffee and cocoa	0.66%	0.75%	0.67%	0.76%	0.82%	0.72%
SD	0.01	0.01	0.01	0.01	0.01	0.01
Budget share SSB	0.00%	2.31%	0.83%	1.95%	4.17%	1.57%
SD	0.00	0.03	0.01	0.02	0.03	0.02
Budget share in water (non-SSBs)	0.02%	0.13%	0.07%	0.13%	0.19%	0.09%
SD	0.00	0.01	0.00	0.00	0.01	0.00
Budget share in alcoholic beverages	0.67%	0.87%	0.64%	0.79%	1.18%	0.81%
SD	0.03	0.03	0.02	0.02	0.03	0.03
Budget share in tobacco	0.70%	0.62%	0.80%	0.64%	0.42%	0.65%
SD	0.03	0.03	0.03	0.03	0.02	0.03
Budget share in clothing and footwear	3.08%	3.53%	3.59%	3.64%	3.37%	3.39%
SD	0.04	0.04	0.04	0.04	0.04	0.04
Budget share in housing, water, electricity, gas and other fuels	13.30%	11.32%	12.38%	11.69%	9.89%	11.95%
SD	0.12	0.10	0.11	0.10	0.09	0.11
Budget share in furnishing, household equipment and others	4.38%	4.86%	4.40%	4.81%	5.39%	4.71%
SD	0.06	0.06	0.05	0.06	0.06	0.06
Budget share in Healthcare	3.57%	3.08%	3.55%	2.89%	2.79%	3.24%
SD	0.08	0.07	0.08	0.06	0.05	0.07
Budget share in transport	11.90%	12.11%	12.51%	12.49%	11.33%	12.04%
SD	0.12	0.12	0.12	0.12	0.11	0.12
Budget share in communication	4.18%	4.30%	4.29%	4.39%	4.22%	4.26%
SD	0.05	0.05	0.05	0.05	0.04	0.05
Budget share in recreation and culture	3.83%	4.01%	4.13%	4.08%	3.81%	3.95%
SD	0.07	0.06	0.06	0.06	0.05	0.06
Budget share in education	1.70%	2.08%	2.06%	2.07%	2.10%	1.96%
SD	0.06	0.06	0.06	0.06	0.05	0.06
Budget share in restaurants and hotels	4.88%	5.41%	5.64%	5.27%	5.30%	5.24%
SD	0.10	0.10	0.10	0.09	0.09	0.10
Budget share in others	8.96%	9.11%	9.37%	9.12%	8.83%	9.06%
SD	0.09	0.08	0.08	0.08	0.08	0.08

*SD* Standard deviation

Low, medium and high SSB consumption are tertiles of households grouped according to positive expenditures on SSB

^a^Positive expenditures include all households that have some expenditures on SSB

^b^“Total” includes all households in the sample (8,632 households)

Table 2 shows budget shares of each of the 16 groups in which the universe of goods and services purchased by Jamaican households was divided. The category of goods or services that concentrate, on average, most of the total expenditure is “Food (consumed at home)” (36%), followed by “Transport” (12%) and “Housing, water, electricity, gas and other fuels” (12%). These three groups are the ones that concentrate the greatest proportion of the total expenditures in all the SSB expenditure groups.

The marginal effects (i.e. the difference in predicted probabilities, in percentage points, across groups) of the GOPM are presented in Table 3. The coefficients in the columns of Table 3 can be interpreted as marginal effects from different probit models jointly estimated. For instance, Column 1 can be interpreted as marginal effects of a probit model on the decision of not having positive expenditures on SSB or having them. Column 2 can be interpreted as the marginal effects of a probit model on the decision of having no expenditures on SSB or having medium or high expenditures on SSB versus having low expenditures on SSB. Column 3 can be interpreted like for Column 2, but now, on the decision of having no expenditures, low or high expenditures on SSB versus having medium expenditures on SSB. Finally, Column 4 shows the marginal effects of having less than high expenditures on SSB (including having none) versus having high expenditures on SSB.

**Table 3:** Marginal effects for the Generalized Ordered Probit model for SSB expenditures

	**(1)**	**(2)**	**(3)**	**(4)**
**VARIABLES**	**No SSB expenditures vs positive SSB expenditures**	**No, medium or high SSB expenditures vs low SSB expenditures**	**No, low or high SSB expenditures vs medium SSB expenditures**	**Less-than-high SSB expenditures vs high SSB expenditures**
Urban household (ref: rural household)	0.023**	-0.003	-0.007	-0.013
	(0.010)	(0.009)	(0.009)	(0.008)
Sex of household (HH) head	-0.012	0.006	0.005	0.001
	(0.010)	(0.009)	(0.009)	(0.008)
Age of HH head	0.009***	-0.001	-0.001	-0.008***
	(0.002)	(0.001)	(0.001)	(0.001)
Age squared of HH head	-0.000***	0.000	0.000	0.000***
	(0.000)	(0.000)	(0.000)	(0.000)
Log of HH size	0.036***	0.005	-0.014	-0.027***
	(0.010)	(0.009)	(0.009)	(0.009)
Proportion of women in HH	-0.011	0.038***	0.003	-0.031**
	(0.016)	(0.015)	(0.015)	(0.014)
Proportion of children in the HH	-0.079***	0.003	0.015	0.061***
	(0.026)	(0.023)	(0.023)	(0.022)
Someone employed in the household (ref: no one employed in the household)	-0.009	-0.009	0.015	0.004
	(0.012)	(0.011)	(0.011)	(0.010)
Log Total HH expenditure	-0.167***	-0.075***	0.035***	0.207***
	(0.006)	(0.005)	(0.005)	(0.005)
Observations	8,760	8,760	8,760	8,760

Standard errors in parentheses, ^***^p < 0.01, ** *p* < 0.05, * *p* < 0.1

The decision of not having SSB expenditures (Column 1) is positively associated with living in urban areas, larger households and with the age of the household head (an extra year is associated to a 0.9 percentage point increase in the probability of not having positive SSB expenditures); and negatively associated with the proportion of children in the households and the logarithm of total household expenditures (for instance, a 10% increase in total household expenditures decreases the probability of not having SSB expenditures by 16.7 percentage points). On the other hand, having high expenditures in SSB (Column 4) is positively associated with the proportion of children in the household and the logarithm of total household expenditures.

Table 4 presents the results of 14 equations of the SURE model. A positive (negative) and statistically significant coefficient for the dichotomous variable showing purchases of SSB means that households in that position increase (decrease) the budget share (i.e. relative allocation) of that category. Hence, a positive and statistically significant coefficient for such a dichotomous variable in the equation of “Food (consumed at home)”, for example, implies that households with positive expenditures on SSB allocate a proportionally larger portion of their total expenditures to food expenditures, compared to all other considered variables that were kept constant. This is what is shown, for instance, in the first line of Table 4.

**Table 4:** SURE estimates for budget shares for total population

Variable	BS for Food (at home)	BS for Tea, Coffee and cocoa	BS for Water (non-SSB)	BS for Alcoholic Beverages	BS for Tobacco	BS for Clothing and Footwear	BS for Housing, Water, electricity, etc	BS for Furnishing, household equipment, etc	BS for Healthcare	BS for Transport	BS for Communication	BS for Recreation and Culture	BS for Education	BS for Restaurants and Hotels
**Positive SSB consumption (ref: No SSB consumption)**	0.0368***	0.0022***	0.0009***	0.0035***	0.0022***	0.0050***	-0.0152***	0.0030**	-0.0030*	-0.0180***	-0.0024**	-0.0029*	-0.0029**	-0.0056**
	**(0.0040)**	**(0.0003)**	**(0.0001)**	**(0.0007)**	**(0.0007)**	**(0.0010)**	**(0.0026)**	**(0.0014)**	**(0.0017)**	**(0.0027)**	**(0.0012)**	**(0.0015)**	**(0.0013)**	**(0.0024)**
Urban household (ref: rural household)	-0.0212***	-0.0019***	0.0005***	-0.0017***	0.0005	-0.0060***	0.0358***	0.0017	-0.0060***	-0.0154***	0.0067***	0.0004	0.0039***	-0.0038*
	(0.0037)	(0.0003)	(0.0001)	(0.0006)	(0.0006)	(0.0009)	(0.0024)	(0.0013)	(0.0016)	(0.0025)	(0.0011)	(0.0014)	(0.0012)	(0.0022)
Sex of the head of the household	0.0118***	0.0000	0.0003**	-0.0083***	-0.0074***	-0.0016*	0.0170***	0.0015	0.0040**	-0.0090***	0.0002	-0.0071***	0.0049***	-0.0064***
	(0.0037)	(0.0003)	(0.0001)	(0.0006)	(0.0006)	(0.0009)	(0.0024)	(0.0013)	(0.0016)	(0.0025)	(0.0011)	(0.0014)	(0.0012)	(0.0022)
Age of the head of the household	0.0014***	0.0001***	-0.0000	-0.0000	-0.0000	-0.0005***	0.0001*	0.0002***	0.0008***	-0.0005***	-0.0001***	-0.0004***	-0.0002***	-0.0007***
	(0.0001)	(0.0000)	(0.0000)	(0.0000)	(0.0000)	(0.0000)	(0.0001)	(0.0000)	(0.0000)	(0.0001)	(0.0000)	(0.0000)	(0.0000)	(0.0001)
Log of household size	0.0087*	-0.0002	-0.0001	-0.0027***	-0.0004	0.0024**	-0.0090***	-0.0042***	0.0046**	-0.0006	-0.0004	0.0003	0.0045***	-0.0047*
	(0.0045)	(0.0003)	(0.0001)	(0.0007)	(0.0008)	(0.0011)	(0.0029)	(0.0016)	(0.0019)	(0.0030)	(0.0013)	(0.0017)	(0.0014)	(0.0026)
Percentage of women in the household	-0.0250***	0.0004	0.0001	-0.0027***	-0.0031***	-0.0025*	0.0243***	0.0057***	0.0055**	0.0018	0.0039**	-0.0039*	0.0004	-0.0146***
	(0.0061)	(0.0004)	(0.0002)	(0.0010)	(0.0010)	(0.0014)	(0.0039)	(0.0022)	(0.0026)	(0.0042)	(0.0018)	(0.0023)	(0.0019)	(0.0036)
Percentage of children in the household	0.0300***	0.0002	-0.0002	0.0021	0.0003	0.0021	-0.0050	-0.0008	-0.0120***	-0.0091	-0.0096***	0.0039	-0.0023	0.0165***
	(0.0105)	(0.0008)	(0.0003)	(0.0018)	(0.0018)	(0.0025)	(0.0068)	(0.0037)	(0.0045)	(0.0072)	(0.0031)	(0.0040)	(0.0033)	(0.0062)
Someone employed in the household (ref: no one employed in the household)	0.0004	0.0002	0.0001	0.0008**	0.0002	0.0013**	-0.0001	-0.0008	-0.0061***	0.0030*	0.0001	-0.0006	-0.0023***	0.0031**
	(0.0023)	(0.0002)	(0.0001)	(0.0004)	(0.0004)	(0.0006)	(0.0015)	(0.0008)	(0.0010)	(0.0016)	(0.0007)	(0.0009)	(0.0007)	(0.0014)
Log Total expenditure	-0.1002***	-0.0013***	0.0003***	-0.0018***	-0.0046***	-0.0015***	-0.0034**	0.0066***	0.0055***	0.0409***	0.0082***	0.0093***	0.0122***	0.0191***
	(0.0025)	(0.0002)	(0.0001)	(0.0004)	(0.0004)	(0.0006)	(0.0016)	(0.0009)	(0.0011)	(0.0017)	(0.0007)	(0.0009)	(0.0008)	(0.0015)
Constant	1.5607***	0.0190***	-0.0031***	0.0372***	0.0687***	0.0750***	0.1410***	-0.0461***	-0.0687***	-0.3548***	-0.0578***	-0.0543***	-0.1336***	-0.1433***
	(0.0309)	(0.0022)	(0.0009)	(0.0052)	(0.0053)	(0.0073)	(0.0199)	(0.0109)	(0.0133)	(0.0212)	(0.0092)	(0.0117)	(0.0098)	(0.0184)
Observations	8,530	8,530	8,530	8,530	8,530	8,530	8,530	8,530	8,530	8,530	8,530	8,530	8,530	8,530
R-squared	0.2051	0.0343	0.0150	0.0349	0.0332	0.0560	0.0501	0.0145	0.0436	0.0783	0.0288	0.0278	0.0429	0.0434

Standard errors in parentheses, ^***^p < 0.01, ** *p* < 0.05, * *p* < 0.1, BS stands for "budget share"

Positive expenditures on SSB are positively and significantly related to a higher share of total expenditures in “Food (consumed at home)” (households spending on SSB allocate, on average, 2.5 percentage points more to food expenditures, keeping everything else constant), “Tea, coffee and cocoa” (0.2 extra percentage points), “Water” (0.1 extra percentage points), “Alcoholic beverages” (0.3 extra percentage points), “Tobacco” (0.2 extra percentage points) and “Clothing and footwear” (0.4 extra percentage points), among others.

On the other hand, the purchase of SSB is significantly and inversely related to the budget share in: “Housing, water, electricity, gas and other fuels” (households spending on SSB allocate 1.7 percentage points less to housing and utilities, keeping everything else constant), “Health” (0.4 percentage points less than households not buying SSB), “Transport” (2 percentage points less than households not buying SSB), “Communication” (0.3 percentage points less than households not buying SSB), “Recreation and culture” (0.3 percentage points less than households not buying SSB), “Education” (0.3 percentage points less than households not buying SSB), and “Restaurants and hotels” (0.5 percentage points less).

Finally, a Breusch-Pagan test for the independence of the errors across equations of the SURE rejected the null hypothesis (Chi-square = 6273.54; *p* < 0.01), showing that errors are correlated across equations.

## Discussions

The World Health Organization recommends a reduced intake of free sugars throughout the life course, limiting it to less than 10% of total energy intake, and preferably to reduce it even further to below 5% of total energy intake for additional benefits [31]. SSB are a major and growing contributor to free sugars’ availability [32, 33], and its consumption is associated to the prevalence of obesity, diabetes, cardiovascular diseases, cancers, and other several health conditions [6–10]. The social and economic costs of SSB consumption include not only direct medical costs, associated with the treatment and care for those illnesses, but crucially, the value of productive and life-quality years lost to diseases and incapacities [4, 34]. Hence, these costs are borne not only by those consuming these goods, but by the society. When this happens (i.e., the consumption of products implies social costs that are larger than the private ones), it is optimal to tax these products and apply other demand reduction policies, such as regulations for marketing, labeling, and the school environment and other settings, in order to decrease their consumption [33–37].

In the case of Jamaica, SSB are not taxed, apart from the general consumption tax on all goods and services (16.5% of the value of goods after duties). No other fiscal measure (e.g., subsidies on water) is in place to disincentivize the consumption of SSB by altering market relative prices. There is no information on the evolution of affordability (i.e. how many hours/days of work-salary are needed to buy one liter of SSB), although it is known that in the region, prices of SSB have suffered a strong decrease when compared to nominal wages [38]. If this is the case for Jamaica (and there are no reasons to believe it is not), it is quite possible that the per capita consumption of SSB has grown over the past years. Therefore, it is highly advisable then to impose taxes as part of a comprehensive package of regulatory policies that could curb such a consumption.

Taxing SSB is a strategy that is being increasingly adopted by both developing and developed countries around the world [39, 40]. Numerous studies have concluded that, because of the existence of healthy, nearly-free substitutes (plain water), taxing SSB does not constitute a financial burden on poorer households, as they usually are elastic goods [39, 40]. In addition, there is evidence that such taxes do not lead to an economic burden in terms of job losses, as the reallocation of expenditures (away from taxed SSB) imply the creation of jobs in other sectors of the economy [39, 40].

Taxation can also bring fiscal revenues that can be used to increase resources devoted to healthcare and/or to promote the consumption of healthy substitutes (plain water). A program to, for instance, provide water dispensers or even unsweetened milk at schools, in both rural and urban areas, could be financed with these revenues.

As for other demand reduction policies, Jamaica has not yet regulated labeling and marketing of SSBs in ways that could effectively reduce consumption of these products [33–37, 41, 42]. National policies to regulate marketing and labeling including the mandatory application of warning labels and the restriction of marketing of products with such warnings have been effective in reducing sales of SSBs and other products excessive in sugars. In Chile, the purchase of calories from products high in sugars, including SSBs, was effectively decreased by 26.7%, after the first year of implementation of their integrated policy system that combined warning labels, marketing restrictions and regulation of the school food environment [43]. The regulation of the school environment including the banning of SSBs from schools has been demonstrated to be one of the most cost-effective policies to reduce obesity along the taxation of SSBs and has been adopted in many countries [37, 44, 45]. Jamaica has taken a step towards this ban, when in January 2021 a gradual elimination of many SSBs was enacted based on their sugar content. However, even after the last and most rigorous threshold for sugar content is enforced in 2023, some SSBs will still be allowed in schools, such as juices and SSBs with less than 2.5 g of sugars per 100 ml [46].

The position of Jamaica among the countries with highest consumption of SSBs and prevalence of obesity worldwide [2, 12], makes imperative the need to adopt and strengthen this integrated set of policies to effectively reduce the demand for and offer of SSBs.

This article has some limitations that should be considered. First, the dataset used in the analyses is relatively old. The survey was collected in 2004–2005 and it is likely that consumption trends may have changed since then. In this regard, results must be considered with caution. Unfortunately, the most recent HES, collected in 2017, is not publicly available. Second, the model estimated by SURE may have endogenous regressors, concretely the variable SSB that can be correlated to the error terms [28]. Hence, no causality is implied in the estimated SURE model. Third, the dataset does not provide any information on physical quantities consumed (units, liters), precluding the possibility of estimating amounts of products or amounts of sugars consumed.

## Conclusions

Households located in rural areas, with smaller numbers of members, with younger heads of the household, with children and with lower total household expenditures were more likely to spend their budget on SSB. This study has also demonstrated that SSB expenditures displaced household expenditures in necessary goods and services, including housing, transport, healthcare and education.

The findings imply that decreasing the amount of budget spent on SSB may have important present and future consequences on poorer households’ human capital accumulation and future incomes.

## Supplementary Information

**Additional file 1:** **Table S1.** COICOP codes by expenditure category. **Table S2. **SURE estimates for budget shares for total population, including households with incomplete expenditure information. **Table S3.** SURE estimates for budget shares for total population, assuming intra-household economies of scale in consumption.

## Data Availability

Data is available from the Statistical Institute of Jamaica (https://statinja.gov.jm/).

## Abbreviations

COICOP: Classification of Individual Consumption According to Purpose
GOPM: Generalized ordered probit model
HES: Household Expenditure Survey
PSU: Primary Sampling Units
OPM: Ordered probit model
SSB: Sugar-sweetened beverages
SURE: Seemingly Unrelated Regression Equations
