# Supplementary material for: Expenditures on sugar-sweetened beverages in Jamaica and its association with household budget allocation
Source: BMC Public Health. 2022 Mar 24;22:580. doi: 10.1186/s12889-022-12959-7 (PMC8943997; doi:10.1186/s12889-022-12959-7)
Supplement: Supplementary file 1 — Additional file 1: Table S1. COICOP codes by expenditure category. Table S2. SURE estimates for budget shares for total population, including households with incomplete expenditure information. Table S3. SURE estimates for budget shares for total population, assuming intra-household economies of scale in consumption. [file 12889_2022_12959_MOESM1_ESM.docx]

**Table S1**. COICOP codes by expenditure category

| ***Categories*** | ***COICOP codes included*** |
| --- | --- |
| **Food and non-alcoholic beverages** | 0111.231009 to 0122.24490800, except those in the "Tea, Coffee and Cocoa", SSB and Non-SSB categories |
| **Tea, Coffee and cocoa** | 0121.23630000; 0121.23630900; 0121.23640000; 0121.23660000; 0121.23911000; 0121.23911100; 0121.23911200; 0121.23913000; 0121.23913100 |
| **Sugar-sweetened beverages (SSB)** | 0122.21400000 to 0122.24490900, except those in the Non SSB category |
| **Non SSB** | 0122.2441; 0122.24410200; 0122.24410200 |
| **Alcoholic Beverages** | 0211.24130000 to 0213.24130390 |
| **Tobacco and narcotics** | 0220.25010000 to 0220.25010900 |
| **Clothing and Footwear** | 0311.26510000 to 0322.87220100 |
| **Housing, Water, electricity, etc.** | 0400.99999999 to 0454.34510000 |
| **Furnishing, household equipment, etc.** | 0511.36990100 to 0562.98000900 |
| **Healthcare** | 0611.35250000 to 0630.93199900 |
| **Transportation** | 0711.49113000 to 0736.64235000 |
| **Communication** | 0810.32510000 to 0830.84200000 |
| **Recreation and Culture** | 0900.00000000 to 0954.38911000 |
| **Education** | 1010.93321000 to 1060.99999999 |
| **Restaurants and Hotels** | 1111.23999190 to 1120.92190400 |
| **Other expenditures** | 1211.38972100 to 1502.50090000 |

**Table S2.** SURE estimates for budget shares for total population, including households with incomplete expenditure information

| Variable | BS for Food (at home) | BS for Tea, Coffee and cocoa | BS for Non SSB | BS for Alcoholic Beverages | BS for Tobacco | BS for Clothing and Footwear | BS for Housing, Water, electricity, etc. | BS for Furnishing, household equipment, etc. | BS for Healthcare | BS for Transport | BS for Communication | BS for Recreation and Culture | BS for Education | BS for Restaurants and Hotels |
| --- | --- | --- | --- | --- | --- | --- | --- | --- | --- | --- | --- | --- | --- | --- |
|  |  |  |  |  |  |  |  |  |  |  |  |  |  |  |
| **Positive SSB consumption (ref: No SSB consumption)** | **0.0366***** | **0.0021***** | **0.0009***** | **0.0035***** | **0.0022***** | **0.0050***** | **-0.0143***** | **0.0031**** | **-0.0032*** | **-0.0180***** | **-0.0022*** | **-0.0026*** | **-0.0027**** | **-0.0047**** |
|  | **(0.0039)** | **(0.0003)** | **(0.0001)** | **(0.0007)** | **(0.0007)** | **(0.0009)** | **(0.0025)** | **(0.0014)** | **(0.0017)** | **(0.0027)** | **(0.0012)** | **(0.0015)** | **(0.0013)** | **(0.0023)** |
| Urban household (ref: rural household) | -0.0237*** | -0.0019*** | 0.0005*** | -0.0019*** | 0.0004 | -0.0062*** | 0.0349*** | 0.0015 | -0.0063*** | -0.0147*** | 0.0067*** | -0.0000 | 0.0041*** | -0.0037* |
|  | (0.0036) | (0.0003) | (0.0001) | (0.0006) | (0.0006) | (0.0009) | (0.0023) | (0.0013) | (0.0016) | (0.0025) | (0.0011) | (0.0014) | (0.0012) | (0.0022) |
| Sex of the head of the household | 0.0127*** | 0.0000 | 0.0003** | -0.0081*** | -0.0073*** | -0.0016* | 0.0171*** | 0.0013 | 0.0040** | -0.0094*** | 0.0005 | -0.0071*** | 0.0048*** | -0.0069*** |
|  | (0.0037) | (0.0003) | (0.0001) | (0.0006) | (0.0006) | (0.0009) | (0.0024) | (0.0013) | (0.0016) | (0.0025) | (0.0011) | (0.0014) | (0.0012) | (0.0022) |
| Age of the head of the household | 0.0014*** | 0.0001*** | -0.0000 | -0.0000 | -0.0000 | -0.0005*** | 0.0001** | 0.0002*** | 0.0008*** | -0.0005*** | -0.0001*** | -0.0004*** | -0.0002*** | -0.0007*** |
|  | (0.0001) | (0.0000) | (0.0000) | (0.0000) | (0.0000) | (0.0000) | (0.0001) | (0.0000) | (0.0000) | (0.0001) | (0.0000) | (0.0000) | (0.0000) | (0.0001) |
| Log household size | 0.0098** | -0.0000 | -0.0001 | -0.0023*** | -0.0002 | 0.0032*** | -0.0073*** | -0.0051*** | 0.0007 | 0.0016 | 0.0004 | -0.0000 | 0.0026** | -0.0026 |
|  | (0.0038) | (0.0003) | (0.0001) | (0.0006) | (0.0007) | (0.0009) | (0.0025) | (0.0013) | (0.0016) | (0.0026) | (0.0011) | (0.0014) | (0.0012) | (0.0023) |
| Percentage of women in the household | -0.0256*** | 0.0004 | 0.0001 | -0.0026*** | -0.0032*** | -0.0023 | 0.0222*** | 0.0059*** | 0.0060** | 0.0010 | 0.0028 | -0.0040* | 0.0009 | -0.0139*** |
|  | (0.0060) | (0.0004) | (0.0002) | (0.0010) | (0.0010) | (0.0014) | (0.0039) | (0.0021) | (0.0026) | (0.0041) | (0.0018) | (0.0023) | (0.0019) | (0.0036) |
| Percentage of children in the household | 0.0285*** | -0.0000 | -0.0003 | 0.0012 | 0.0001 | 0.0003 | -0.0075 | 0.0004 | -0.0053 | -0.0129* | -0.0109*** | 0.0044 | 0.0005 | 0.0124** |
|  | (0.0097) | (0.0007) | (0.0003) | (0.0016) | (0.0017) | (0.0023) | (0.0062) | (0.0034) | (0.0042) | (0.0067) | (0.0029) | (0.0037) | (0.0031) | (0.0058) |
| Someone employed in the household (ref: no one employed in the household) | 0.0024 | 0.0005 | 0.0000 | 0.0021*** | 0.0000 | 0.0031*** | -0.0019 | 0.0003 | -0.0103*** | 0.0034 | -0.0027** | -0.0011 | -0.0030** | 0.0066** |
|  | (0.0044) | (0.0003) | (0.0001) | (0.0007) | (0.0007) | (0.0010) | (0.0028) | (0.0015) | (0.0019) | (0.0030) | (0.0013) | (0.0016) | (0.0014) | (0.0026) |
| Log Total expenditure | -0.1032*** | -0.0014*** | 0.0002*** | -0.0019*** | -0.0045*** | -0.0017*** | -0.0048*** | 0.0064*** | 0.0051*** | 0.0421*** | 0.0079*** | 0.0087*** | 0.0125*** | 0.0184*** |
|  | (0.0024) | (0.0002) | (0.0001) | (0.0004) | (0.0004) | (0.0006) | (0.0015) | (0.0008) | (0.0010) | (0.0017) | (0.0007) | (0.0009) | (0.0008) | (0.0014) |
| Constant | 1.5963*** | 0.0197*** | -0.0029*** | 0.0379*** | 0.0685*** | 0.0756*** | 0.1597*** | -0.0443*** | -0.0595*** | -0.3687*** | -0.0518*** | -0.0465*** | -0.1363*** | -0.1379*** |
|  | (0.0302) | (0.0022) | (0.0009) | (0.0050) | (0.0051) | (0.0072) | (0.0195) | (0.0107) | (0.0130) | (0.0208) | (0.0090) | (0.0114) | (0.0097) | (0.0179) |
|  |  |  |  |  |  |  |  |  |  |  |  |  |  |  |
| Observations | 8,760 | 8,760 | 8,760 | 8,760 | 8,760 | 8,760 | 8,760 | 8,760 | 8,760 | 8,760 | 8,760 | 8,760 | 8,760 | 8,760 |
| R-squared | 0.2201 | 0.0357 | 0.0147 | 0.0355 | 0.0337 | 0.0567 | 0.0479 | 0.0145 | 0.0424 | 0.0818 | 0.0282 | 0.0264 | 0.0448 | 0.0424 |
| Standard errors in parentheses  *** p<0.01, ** p<0.05, * p<0.1 |  |  |  |  |  |  |  |  |  |  |  |  |  |  |
| BS stands for "budget share" |  |  |  |  |  |  |  |  |  |  |  |  |  |  |

**Table S3**. SURE estimates for budget shares for total population, assuming intra-household economies of scale in consumption

| **Variable** | **BS for Food (at home)** | **BS for Tea, Coffee and cocoa** | **BS for Non SSB** | **BS for Alcoholic Beverages** | **BS for Tobacco** | **BS for Clothing and Footwear** | **BS for Housing, Water, electricity, etc.** | **BS for Furnishing, household equipment, etc.** | **BS for Healthcare** | **BS for Transport** | **BS for Communication** | **BS for Recreation and Culture** | **BS for Education** | **BS for Restaurants and Hotels** |
| --- | --- | --- | --- | --- | --- | --- | --- | --- | --- | --- | --- | --- | --- | --- |
| **Positive SSB consumption (ref: No SSB consumption)** | **0.0252***** | **0.0019***** | **0.0008***** | **0.0031***** | **0.0019***** | **0.0041***** | **-0.0177***** | **0.0018** | **-0.0037**** | **-0.0205***** | **-0.0034***** | **-0.0038**** | **-0.0032**** | **-0.0067***** |
|  | **(0.0039)** | **(0.0003)** | **(0.0001)** | **(0.0007)** | **(0.0007)** | **(0.0009)** | **(0.0026)** | **(0.0014)** | **(0.0017)** | **(0.0027)** | **(0.0012)** | **(0.0015)** | **(0.0013)** | **(0.0023)** |
| Urban household (ref: rural household) | -0.0225*** | -0.0019*** | 0.0005*** | -0.0018*** | 0.0004 | -0.0059*** | 0.0373*** | 0.0023* | -0.0060*** | -0.0156*** | 0.0070*** | 0.0002 | 0.0038*** | -0.0042* |
|  | (0.0036) | (0.0003) | (0.0001) | (0.0006) | (0.0006) | (0.0009) | (0.0023) | (0.0013) | (0.0016) | (0.0025) | (0.0011) | (0.0014) | (0.0012) | (0.0022) |
| Sex of the head of the household | 0.0106*** | 0.0000 | 0.0003*** | -0.0082*** | -0.0073*** | -0.0017* | 0.0189*** | 0.0017 | 0.0038** | -0.0091*** | 0.0003 | -0.0072*** | 0.0048*** | -0.0066*** |
|  | (0.0036) | (0.0003) | (0.0001) | (0.0006) | (0.0006) | (0.0009) | (0.0023) | (0.0013) | (0.0016) | (0.0025) | (0.0011) | (0.0014) | (0.0012) | (0.0022) |
| Age of the head of the household | 0.0013*** | 0.0001*** | -0.0000 | -0.0000 | -0.0000 | -0.0005*** | 0.0002** | 0.0002*** | 0.0008*** | -0.0005*** | -0.0001*** | -0.0004*** | -0.0002*** | -0.0007*** |
|  | (0.0001) | (0.0000) | (0.0000) | (0.0000) | (0.0000) | (0.0000) | (0.0001) | (0.0000) | (0.0000) | (0.0001) | (0.0000) | (0.0000) | (0.0000) | (0.0001) |
| Log household size | 0.0035 | -0.0002 | -0.0001 | -0.0029*** | -0.0006 | 0.0021** |  |  | 0.0040** | -0.0026 |  | -0.0004 | 0.0040*** | -0.0066** |
|  | (0.0040) | (0.0003) | (0.0001) | (0.0007) | (0.0007) | (0.0010) |  |  | (0.0019) | (0.0030) |  | (0.0017) | (0.0014) | (0.0026) |
| Percentage of women in the household | -0.0087 | 0.0007 | 0.0001 | -0.0020** | -0.0025** | -0.0019 |  |  | 0.0068*** | 0.0062 |  | -0.0020 | 0.0016 | -0.0097*** |
|  | (0.0055) | (0.0004) | (0.0002) | (0.0010) | (0.0010) | (0.0014) |  |  | (0.0026) | (0.0041) |  | (0.0023) | (0.0019) | (0.0035) |
| Percentage of kids in the household | 0.0195** | 0.0000 | -0.0002 | 0.0018 | 0.0001 | 0.0023 |  |  | -0.0125*** | -0.0104 |  | 0.0035 | -0.0030 | 0.0144** |
|  | (0.0094) | (0.0007) | (0.0003) | (0.0017) | (0.0018) | (0.0024) |  |  | (0.0044) | (0.0070) |  | (0.0039) | (0.0033) | (0.0060) |
| Someone employed in the household (ref: no one employed in the household) | 0.0025 | 0.0002 | 0.0001 | 0.0009** | 0.0003 | 0.0014** | -0.0031** | -0.0021*** | -0.0058*** | 0.0036** | -0.0002 | -0.0004 | -0.0021*** | 0.0038*** |
|  | (0.0022) | (0.0002) | (0.0001) | (0.0004) | (0.0004) | (0.0005) | (0.0013) | (0.0007) | (0.0010) | (0.0016) | (0.0006) | (0.0009) | (0.0007) | (0.0014) |
| Log Total expenditure | -0.0972*** | -0.0012*** | 0.0002*** | -0.0017*** | -0.0044*** | -0.0014** | -0.0031* | 0.0065*** | 0.0056*** | 0.0410*** | 0.0082*** | 0.0094*** | 0.0122*** | 0.0191*** |
|  | (0.0024) | (0.0002) | (0.0001) | (0.0004) | (0.0004) | (0.0006) | (0.0016) | (0.0009) | (0.0010) | (0.0017) | (0.0007) | (0.0009) | (0.0008) | (0.0014) |
| Constant | 1.5247*** | 0.0184*** | -0.0031*** | 0.0360*** | 0.0671*** | 0.0725*** | 0.1393*** | -0.0450*** | -0.0693*** | -0.3570*** | -0.0597*** | -0.0558*** | -0.1328*** | -0.1438*** |
|  | (0.0302) | (0.0022) | (0.0009) | (0.0051) | (0.0052) | (0.0072) | (0.0197) | (0.0107) | (0.0132) | (0.0210) | (0.0090) | (0.0116) | (0.0097) | (0.0182) |
|  |  |  |  |  |  |  |  |  |  |  |  |  |  |  |
| Observations | 8,530 | 8,530 | 8,530 | 8,530 | 8,530 | 8,530 | 8,530 | 8,530 | 8,530 | 8,530 | 8,530 | 8,530 | 8,530 | 8,530 |
| R-squared | 0.2048 | 0.0333 | 0.0155 | 0.0345 | 0.0329 | 0.0549 | 0.0458 | 0.0119 | 0.0435 | 0.0792 | 0.0263 | 0.0277 | 0.0428 | 0.0430 |
| Standard errors in parentheses  *** p<0.01, ** p<0.05, * p<0.1 |  |  |  |  |  |  |  |  |  |  |  |  |  |  |
| BS stands for "budget share" |  |  |  |  |  |  |  |  |  |  |  |  |  |  |
